# Disease-Toxicant Interactions in Manganese Exposed Huntington Disease Mice: Early Changes in Striatal Neuron Morphology and Dopamine Metabolism

**DOI:** 10.1371/journal.pone.0031024

**Published:** 2012-02-17

**Authors:** Jennifer L. Madison, Michal Wegrzynowicz, Michael Aschner, Aaron B. Bowman

**Affiliations:** 1 Department of Pharmacology, Vanderbilt University Medical Center, Nashville, Tennessee, United States of America; 2 Department of Neurology, Vanderbilt University Medical Center, Nashville, Tennessee, United States of America; 3 Department of Pediatrics, Vanderbilt University Medical Center, Nashville, Tennessee, United States of America; 4 Vanderbilt University Kennedy Center for Research on Human Development, Vanderbilt University Medical Center, Nashville, Tennessee, United States of America; 5 Center for Molecular Neuroscience, Vanderbilt University Medical Center, Nashville, Tennessee, United States of America; 6 Center in Molecular Toxicology, Vanderbilt University Medical Center, Nashville, Tennessee, United States of America; University of Cambridge, United Kingdom

## Abstract

YAC128 Huntington's disease (HD) transgenic mice accumulate less manganese (Mn) in the striatum relative to wild-type (WT) littermates. We hypothesized that Mn and mutant *Huntingtin* (*HTT*) would exhibit gene-environment interactions at the level of neurochemistry and neuronal morphology. Twelve-week-old WT and YAC128 mice were exposed to MnCl_2_-4H_2_O (50 mg/kg) on days 0, 3 and 6. Striatal medium spiny neuron (MSN) morphology, as well as levels of dopamine (DA) and its metabolites (which are known to be sensitive to Mn-exposure), were analyzed at 13 weeks (7 days from initial exposure) and 16 weeks (28 days from initial exposure). No genotype-dependent differences in MSN morphology were apparent at 13 weeks. But at 16 weeks, a genotype effect was observed in YAC128 mice, manifested by an absence of the wild-type age-dependent increase in dendritic length and branching complexity. In addition, genotype-exposure interaction effects were observed for dendritic complexity measures as a function of distance from the soma, where only YAC128 mice were sensitive to Mn exposure. Furthermore, striatal DA levels were unaltered at 13 weeks by genotype or Mn exposure, but at 16 weeks, both Mn exposure and the HD genotype were associated with quantitatively similar reductions in DA and its metabolites. Interestingly, Mn exposure of YAC128 mice did not further decrease DA or its metabolites versus YAC128 vehicle exposed or Mn exposed WT mice. Taken together, these results demonstrate Mn-HD disease-toxicant interactions at the onset of striatal dendritic neuropathology in YAC128 mice. Our results identify the earliest pathological change in striatum of YAC128 mice as being between 13 to 16 weeks. Finally, we show that mutant *HTT* suppresses some Mn-dependent changes, such as decreased DA levels, while it exacerbates others, such as dendritic pathology.

## Introduction

Huntington's disease (HD) is an autosomal dominant inherited disease caused by expansion of a CAG triplet-repeat within the first exon of the *Huntingtin (HTT)* gene [1]. The primary neuropathology in HD is loss of striatal, followed by cortical neurons [2]. HD causes proliferative and/or degenerative changes in striatal medium spiny neurons (MSNs) as evidenced by post-mortem studies [3], [4], [5]. Changes in MSN morphology precede gross striatal neuron loss [5]. Analysis of MSN morphology can identify early neuropathology prior to death of MSNs and further loss of striatal neurons. Although CAG repeat length is inversely related to the age of disease-onset in humans, it only accounts for about 60% of the variability in disease onset [6], [7], [8], [9], [10]. Environmental and/or other genetic factors account for the remaining variability in disease onset and therefore may accelerate or slow the age of disease onset and progression [11], [12], [13], [14]. Metal toxicity and disruption in metal homeostasis (e.g. iron, copper, zinc and manganese (Mn)) has been associated with many neurodegenerative diseases including Parkinson's disease, Huntington's disease and Alzheimer's disease [15], [16], [17]. Here we explore the possibility that exposure to Mn may modulate HD pathophysiology.

Mn is an essential trace metal that is critical for many physiological processes including reproduction, formation of connective tissue and bone and normal brain function including neurotransmitter synthesis and metabolism [18], [19], [20], [21]. Exposure to high Mn levels causes neurotoxicity, especially in brain regions where Mn preferentially accumulates including the globus pallidus, striatum, substantia nigra and the subthalamic nucleus [22], [23]. Rodent studies examining MSN morphology following Mn exposure found a decrease in total dendritic length and spine number [24]. Striatal dopamine (DA) neurotransmitter levels are decreased in some, but not all, animal models of Mn neurotoxicity [25], [26], [27], [28], [29], [30], [31], [32]. An increase in brain Mn levels is also known to cause motor dysfunction in humans, non-human primates and rodents [22], [23], [33], [34], [35], [36]. Since the basal ganglia, including the striatum are a common target for both HD neuropathology and Mn accumulation, this provides the opportunity to observe a disease-toxicant interaction.

Data previously published by our group shows that following Mn exposure the YAC128 HD mouse model exhibits decreased striatal accumulation of Mn relative to wild-type (WT) mice [37]. This phenomenon was selectively found in the corpus striatum, the most vulnerable brain region in HD, months before detectable neurodegenerative pathological changes [37], [38]. *In vitro* work showed that a striatal cell line expressing mutant *HTT* (STHdh^Q111/Q111^) also accumulated less Mn upon exposure, exhibit a basal Mn deficiency under normal culture conditions and were resistant to Mn cytotoxicity relative to wild-type striatal cells (STHdh^Q7/Q7^) [37], [39], [40]. Based on these studies, we hypothesized that Mn exposure could suppress striatal phenotypes in the YAC128 mice caused by an HD-dependent Mn handling deficit. Furthermore, we hypothesized that YAC128 mice would exhibit diminished Mn toxicity phenotypes. An alternative hypothesis was also considered, that expression of the toxic mutant HTT protein in YAC128 animals could interact with striatal Mn neurotoxicity to elicit or enhance changes in dendritic morphology or dopamine neurochemistry. Here we test these two alternative hypotheses by examining MSN architecture and striatal DA content following Mn exposure in WT and YAC128 mice at 3 months of age. This is the age when the earliest detectable motor dysfunction has been reported and is also the age at which we previously [37], [38] found a defect in striatal Mn accumulation.

## Materials and Methods

### Chemical Reagents

Osmium tetroxide and glutaraldehyde were obtained from Electron Microscopy Sciences (Hatfield, PA), manganese chloride (MnCl_2_) from Alfa Aesar (Ward Hill, MA), paraformaldehyde from Fisher Scientific (Pittsburgh, PA), Phosphate Buffered Saline (PBS) from Mediatech Inc. (Manassas, VA), isoflurane from Phoenix Pharmaceutical Inc. (St. Joseph, MO), and all other chemicals were obtained from Sigma Chemical Company (St. Louis, MO).

### Animal Housing and Manganese Exposure

The FVB-Tg (YAC128)53Hay/J mouse line (YAC128) was purchased from JAX (#004938, Bar Harbor, ME) [38]. All animal exposure protocols were approved by the Vanderbilt University Medical Center Institutional Animal Care and Use Committee (IACUC) and strictly adhered to in order to minimize pain. All exposure and procedures followed NIH laboratory animal care and use guidelines. Genotyping of the mice and confirmation of a consistent CAG-triplet repeat length in mutant animals was carried out by PCR according to a previously published protocol (#004938; from JAX) [38]. Mice were distributed into exposure groups across multiple litters and the gender in each of the groups was balanced. The Mn exposure paradigm was adapted from the previously published protocol [37], [41]. Male C57BL/6 mice, 7–9 months old, exposed to Mn using this protocol showed a significant increase in striatal Mn levels without significant motor impairment on experimental day 7, 24 hours following the last Mn exposure. Twelve-week old mice were subcutaneously (s.c.) injected at the hind leg with vehicle (water) or MnCl_2_-4H_2_O (50 mg/kg) on experimental day 0, 3, and 6. Mice were subsequently used for either inductively coupled plasma mass spectrometry (ICP-MS) analysis of striatal metals, Golgi impregnation for analysis of neuron morphology or striatal neurochemistry analysis.

### Striatal Manganese Levels

Mice were decapitated on experimental day 7 (13 weeks, 7 mice per group) or 28 (16 weeks, 5 mice per group) and the striatum was rapidly dissected. The striatum was then placed in liquid nitrogen and stored at −80°C. Samples were dried in glass vials on a heat block at 100°C for 3 days underneath a beaker turned upside down and digested with 65–70% nitric acid for 3 hours at 90°C. To each sample, 10 mL of 1% nitric acid was added. Next, samples were filtered (Arcodisc, 0.45 µm HT Tuffryn membrane) and submitted for ICP-MS analysis. A Perkin-Elmer ELAN DRC II ICP-MS was calibrated and verified before reading the blank (1% nitric acid). 50 µL of In-115 at a concentration of 50 µg/L was added to each sample as an internal control. Analysis of each sample was read with the following settings: nebulizer flow 0.9–1 L/min, radio frequency power 1,250–1,300 W, plasma gas flow 15 L/min, lens voltage 6.5–7.5 V, auto lens on, 3 replicates per reading, 100 ms dwell time per atomic mass unit, 1000 ms integration time with peak hopping scan mode.

### Golgi Impregnation

In this study we use the single section rapid Golgi method that was optimized to stain MSNs [42], [43]. This method produced high quality staining without any breaks in staining or blebbing along the dendrites (data not shown). In brief, on experimental day 7 (13 weeks, 4–5 mice per group) or day 28 (16 weeks, 5 mice per group) the mice were deeply anesthetized with isoflurane, and transcardially perfused with 15 mL 0.1 M phosphate buffer (PB) followed by 40 ml of 2% paraformaldehyde and 2.5% glutaraldehyde in 0.1 M PB. The brains were removed from the skulls and post-fixed for 3 hours at room temperature. Vibratome sections were cut in the coronal plane at 150 µm. The single-section Golgi silver impregnation method was previously described [42], [43]. The sections were incubated in 1% osmium tetroxide for 20–40 minutes, transferred to 3.5% potassium dichromate and incubated overnight. The sections were then incubated in silver nitrate between two slides for 4–6 hours. Sections were washed in water, mounted on 0.5% gelatin coated slides, dehydrated and cleared and coverslipped.

### Neuron Reconstruction and Analysis

Golgi-impregnated striatal medium spiny neurons were reconstructed under 60× magnification using the NeuroLucida (MicroBrightfield, Inc., Williston, VT) system. One researcher, blinded to genotype and exposure conditions, selected, traced and analyzed the neurons. Four to six neurons were traced per animal and were selected for tracing based on the following strict criteria: location in the striatum (Bregma +0.14 mm to +0.86 mm), soma and dendrites must be fully impregnated without beading or breaks in staining along the dendrite, and must have at least 2 primary dendrites and reach the 3^rd^ branch order. Neurons were analyzed with NeuroExplorer (MicroBrightfield, Inc., Williston, VT). Morphometric characteristics were either averaged over the entire neuron (total neuron measures) or analyzed as a function of distance from the soma. Dendritic length and spine number are summed over the entire dendritic tree to give total dendritic length and total spine number, respectively. Total spine density is the number of spines per 10 µm. The point of each dendrite branching is termed a node. Therefore, the total number of nodes is the total number of branch points summed across the dendritic tree. The total number of ends refers to the number of dendrite endings. Concentric circles of 10 µm radiating outward from the center of the soma were used to analyze morphometric features as a function of distance from the soma (Sholl analysis) [44]. Branching complexity was measured by tallying the number of dendrites that cross each of the concentric circles (intersections). The space in between each of the concentric circles creates a shell in which morphologic features, such as dendrite length, spine number and spine density were measured. This information provides a more detailed view of neuron morphology.

### Dopamine neurochemistry

Mice were euthanized at week 13 or 16 by decapitation. The striatum was rapidly dissected, placed in liquid nitrogen and stored at −80°C. Samples were submitted to the Center for Molecular Neuroscience/Kennedy Center Neurochemistry Core Lab at Vanderbilt University for measurement of dopamine and related metabolites (n = 4–6 mice per group) by high-performance liquid chromatography (HPLC).

### Data Analysis

Univariate, multivariate and repeated measures analysis of variance (ANOVA) followed by post-hoc analysis using Fisher's LSD multiple comparison test were performed using PASW Statistics 18 (SPSS, Inc, Chicago, IL). Grubb's outlier test was used prior to statistical analysis to find and eliminate outliers in data with the significance of alpha set to *p*<0.05. Striatal Mn levels, total neuron measures and striatal neurochemistry were assessed by multivariate ANOVA with genotype, exposure and age (when appropriate) as fixed factors. Genotype, exposure, age (when appropriate) and radius were assigned as fixed factors in the analysis where neuron morphologic characteristics were measured as a function of distance from the soma. When ANOVA results were significant, *post-hoc* analysis of all pairwise combinations was used to determine statistical differences between exposure groups.

## Results

### YAC128 mice accumulate less Mn in the striatum immediately following exposure

**Figure 1:**
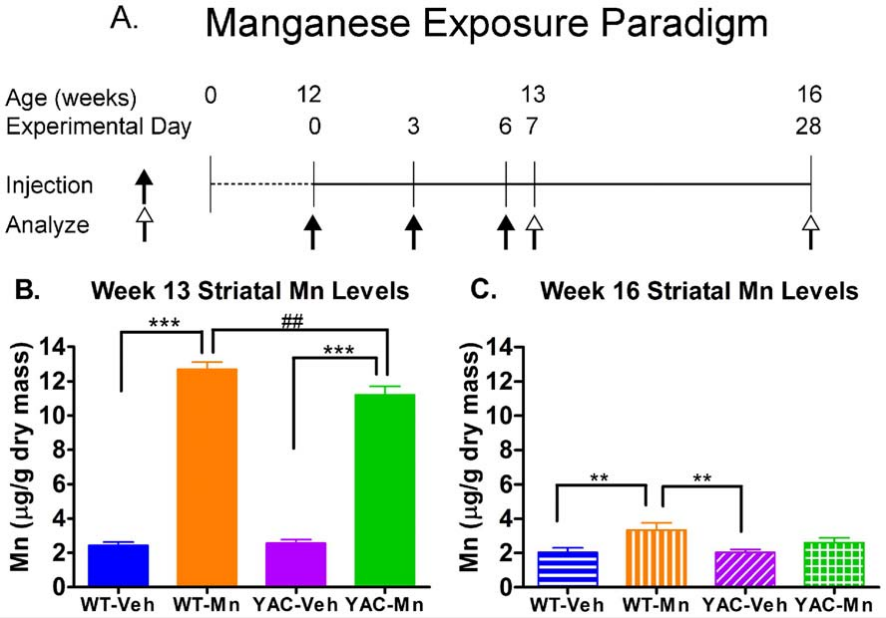
Striatal Mn elevated in both genotypes at 13 but only in WT at week 16. **A**) Mn exposure paradigm. 12 week-old WT and YAC128 mice were exposed to MnCl_2_-4H_2_O (50 mg/kg s.c.) or vehicle (filled arrow) on days 0, 3, 6, and were sacrificed (open arrow) on experimental day 7 (13 weeks) or day 28 (16 weeks) for striatal Golgi impregnation or neurochemistry analysis. **B**) Striatal Mn levels, measured by ICP-MS, at week 13 (n = 7) are significantly higher in Mn exposed mice compared to their vehicle controls at week 13. There is a genotype×exposure interaction whereby significantly less Mn accumulated in the YAC128-Mn exposed group compared to WT-Mn exposed mice. **C**) At week 16 (n = 5), Mn levels dropped, but a exposure effect still remains with Mn levels significantly higher in WT mice. Average striatal Mn concentrations +/− SEM. Significant results of *post-hoc* t-tests: **p<0.01, ^##^p<0.005 and ***p<0.001.

The striatum represents a target region for Mn neurotoxicity and also degeneration in HD [2], [3], [5], [22], [23]. Accordingly, studies were carried out to address the effect of mutant *HTT* and Mn toxicity on striatal MSN morphology and striatal neurotransmitter content in 13 and 16 week old YAC128 and WT mice. Twelve-week old mice were exposed to vehicle (Veh, s.c.) or Mn (50 mg/kg MnCl_2_-4H_2_O, s.c.) on experimental day 0, 3 and 6. Mice were sacrificed on experimental day 7 (13 weeks postnatal) or day 28 (16 weeks postnatal) (Figure 1A). Mice were subsequently used for either measurement of striatal metals by ICP-MS, striatal neurochemical analysis or Golgi impregnation for analysis of striatal MSN morphology.

Our first objective was to measure striatal Mn concentrations by ICP-MS 24 hours (experimental day 7, 13 weeks old) and 3 weeks (experimental day 28, 16 weeks old) following the last Mn injection. At 13 weeks, levels of striatal Mn (Figure 1B) were significantly increased in both Mn exposed WT and YAC128 mice (F_(1,23)_ = 783.43, *p*<0.0001). There was a significant genotype×exposure interaction with YAC128 mice accumulating significantly lower levels of striatal Mn compared to WT (F_(1,23)_ = 5.33, *p*<0.05), corroborating graphite furnace atomic absorbance spectroscopy (GFAAS) results in an independent study [37]. At 16 weeks, (Figure 1C) this exposure effect persisted (F_(1,16)_ = 9.19, *p*<0.01) and Mn levels in the striatum of WT mice were dramatically reduced, but remained significantly elevated compared to vehicle injected mice (p<0.01), but there was no difference in striatal Mn levels between WT-Vehicle (WT-Veh), YAC128-Veh and YAC128-Mn groups at 16 weeks. In addition to Mn, other metals were simultaneously analyzed by ICP-MS in the striatum of these mice. Striatal levels of copper (Cu) and zinc (Zn) and other metals showed no significant differences across the exposure groups at either time point (data not shown). These results demonstrate that the sub-acute Mn exposure paradigm can significantly and specifically elevate striatal Mn levels immediately following exposure. Excess Mn was completely eliminated from YAC128 striatum by 3 weeks post exposure, however WT mice still had slightly elevated levels of striatal Mn.

### Genotype influences neuron morphology at 16 weeks

**Figure 2:**
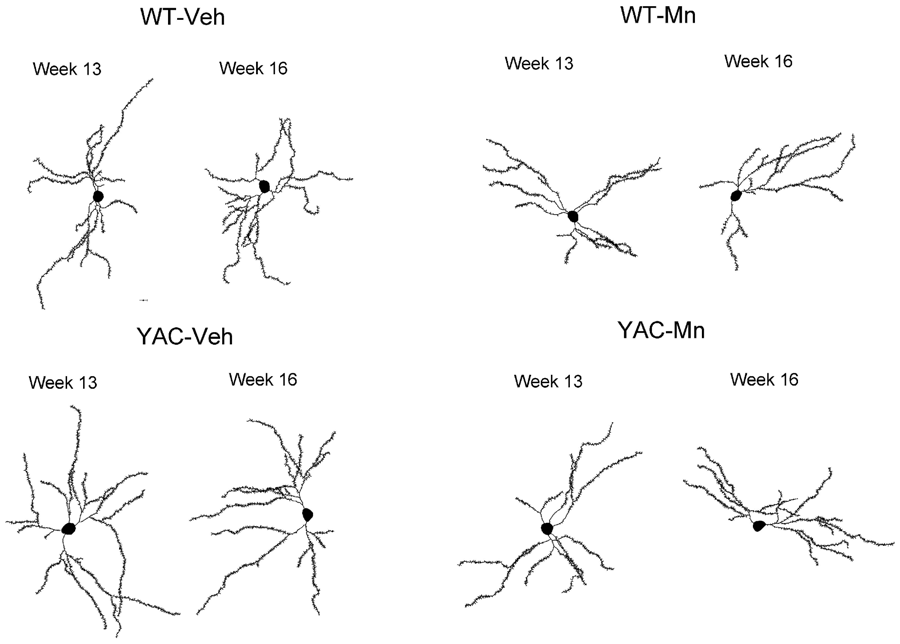
Representative reconstructions of striatal MSN in week 13 and 16 mice. Mice were exposed on day 0, 3, 6 with vehicle or MnCl_2_-4H_2_O (50 mg/kg s.c.), sacrificed on experimental day 7 (week 13, n = 4–5) or 28 (week 16, n = 5) and the striatum was processed for Golgi impregnation. Neurons were traced and analyzed using Neurolucida and Neuroexplorer, respectively (NeuroBrightField, Inc). Representative neurons shown were selected to be within 5% of the mean total dendritic length of each exposure group.

Representative reconstructions of Golgi-impregnated MSNs are shown in Figure 2. MSN morphology was unaffected by genotype or Mn exposure at week 13 (Table 1, Figure S1), however changes in neuron morphology were observed at week 16. There was a significant main effect of genotype at 16 weeks on total dendritic length (*p*<0.01), total spine density (*p*<0.01) and number of dendrite endings (*p*<0.05) (Table 2, Figure S1). YAC128 mice in both the Veh and Mn exposure groups exhibited shorter total dendritic lengths compared to WT-Veh MSNs. YAC128 mice as a group also had higher total spine densities and reduced numbers of dendritic endings than WT mice. These data consistently show that genotype plays a significant role in overall morphological differences observed in MSNs at 16 weeks, while no significant exposure or genotype×exposure interaction effects were seen at this gross level of analysis.

**Table 1:** Two-way ANOVA statistics for total dendritic measures at 13 weeks.

	Genotype	Exposure	Genotype×Exposure
Total Dendritic Length	F(1,93) = 0.125	F(1,93) = 0.287	F(1,93) = 1.855
	p = 0.724	p = 0.593	p = 0.176
Total Spine Density	F(1,93) = 0.001	F(1,93) = 1.831	F(1,93) = 1.00
	p = 0.978	p = 0.179	p = 0.320
Total Spine Number	F(1,93) = 0.143	F(1,93) = 0.002	F(1,93) = 0.902
	p = 0.707	p = 0.966	p = 0.345
Soma Area	F(1,93) = 0.602	F(1,93) = 0.308	F(1,93) = 0.126
	p = 0.440	p = 0.581	p - 0.724
Total Nodes	F(1,93) = 0.019	F(1,93) = 0.514	F(1,93) = 1.817
	p = 0.892	p = 0.475	p = 0.181
Total Ends	F(1,93) = 0.154	F(1,93) = 0.178	F(1,93) = 1.687
	p = 0.696	p = 0.674	p = 0.197

**Table 2:** Two-way ANOVA statistics for total dendritic measures at 16 weeks.

	Genotype	Exposure	Genotype×Exposure
Total Dendritic Length	F(1,109) = 7.503	F(1,109) = 0.669	F(1,109) = 0.000
	**p = 0.007**	p = 0.405	p = 0.986
Total Spine Density	F(1,109) = 7.993	F(1,109) = 0.033	F(1,109) = 0.113
	**p = 0.006**	p = 0.857	p = 0.737
Total Spine Number	F(1,109) = 0.155	F(1,109) = 0.136	F(1,109) = 0.027
	p = 0.649	p = 0.713	p = 0.869
Soma Area	F(1,109) = 3.228	F(1,109) = 0.324	F(1,109) = 0.000
	p = 0.075	p = 0.570	p = 0.998
Total Nodes	F(1,109) = 2.549	F(1,109) = 0.218	F(1,109) = 1.695
	p = 0.113	p = 0.641	p = 0.196
Total Ends	F(1,109) = 4.058	F(1,109) = 0.026	F(1,109) = 1.668
	**p = 0.046**	p = 0.871	p = 0.199

### Morphometric measures as a function of distance from soma are influenced by genotype and Mn exposure

**Figure 3:**
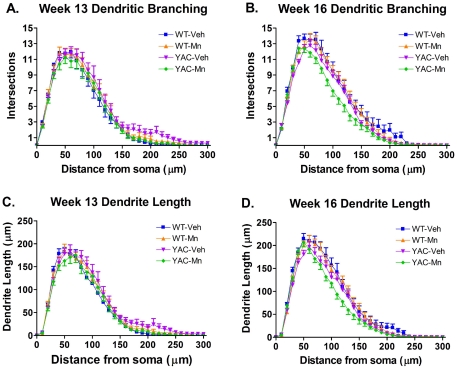
YAC128-Mn exposed mice have decreased branching complexity at 16 weeks. Morphological characteristics of MSNs were assessed using the Sholl method. Concentric rings at 10 µm intervals were centered at the soma and intersections of dendrites at each radius were counted to determine branching complexity. **A**) Dendritic branching complexity is not significantly different between groups at 13 weeks. **B**) Main effects of distance×genotype and distance×genotype×exposure for dendritic branching complexity were observed only at 16 weeks. **C**) Dendrite length as a function of distance from the soma is unaltered at 13 weeks. **D**) At 16 weeks, dendrite length as a function of distance from the soma is subject to distance×genotype and distance×genotype×exposure effects.

Several measures of neuron morphology as a function of distance from the soma were examined in order to gain a detailed understanding of the MSN morphology in these mice. Dendritic arborization is an important morphological characteristic that determines how the neuron integrates synaptic inputs. Sholl analysis measures branching complexity of the neuron by tallying the number of dendrites that intersect a series of evenly spaced concentric circles centered at the soma [44]. At 13 weeks, there was a significant effect of distance on dendritic branching (as expected for Sholl analysis), but no significant interactions between distance×genotype, distance×exposure, or three-way interaction effects (Figure 3A and Table 3). At 16 weeks (Figure 3B and Table 4) there was a significant effect of distance×genotype (*p* = 0.01) and as well as a significant interaction effect between Mn exposure and genotype (distance×genotype×exposure interaction effect, *p*<0.01). *Post-hoc* analysis revealed that the disease-toxicant interaction effect is strongly driven by a difference in branching complexity between WT-Veh and YAC-Mn mice at 16 weeks (*p*<0.05), though trending differences between YAC-Mn and both WT-Mn and YAC-Veh also contributed to the overall significance of the Mn exposure×genotype interaction. These data strongly suggest that the combined effect of Mn exposure and the HD mutant genotype caused a greater decrease in dendritic branching complexity than either factor alone.

**Table 3:** Repeated measures two-way ANOVA statistics for dendritic measures as a function of distance from the soma at 13 weeks.

	Distance×Genotype	Distance×Exposure	Distance×Genotype×Exposure
Sholl	F(39, 3627) = 0.877	F(39, 3627) = 0.177	F(39, 3627) = 0.510
	p = 0.687	p = 1.000	p = 0.995
Dendritic Length	F(39, 3627) = 1.013	F(39, 3627) = 0.211	F(39, 3627) = 0.772
	p = 0.448	p = 1.000	p = 0.845
Spine Number	F(39, 3627) = 0.933	F(39, 3627) = 0.470	F(39, 3627) = 0.741
	p = 0.589	p = 0.998	p = 0.881
Spine Density	F(17, 1581) = 0.299	F(17, 1581) = 0.632	F(17, 1581) = 0.825
	p = 0.997	p = 0.869	p = 0.665

**Table 4:** Repeated measures two-way ANOVA statistics for dendritic measures as a function of distance from the soma at 16 weeks.

	Distance×Genotype	Distance×Exposure	Distance×Genotype×Exposure
Sholl	F(30,2370) = 1.709	F(30,2370) = 0.558	F(30,2370) = 1.713
	**p = 0.0095**	p = 0.975	**p = 0.0093**
Dendritic Length	F(30,2370) = 2.264	F(30,2370) = 0.730	F(30,2370) = 1.613
	**p = 0.0001**	p = 0.8571	**p = 0.0187**
Spine Number	F(30,2370) = 1.717	F(30,2370) = 0.649	F(30,2370) = 3.001
	**p = 0.0090**	p = 0.9287	**p<0.0001**
Spine Density	F(17,1853) = 1.733	F(17,1853) = 1.890	F(17,1853) = 1.513
	**p = 0.0315**	**p = 0.0151**	p = 0.0812

We also examined dendrite length, spine number and spine density as a function of distance from the soma as further measures of dendritic arborization and synaptic integration. These morphological characteristics of dendrites were measured in each shell of the concentric circles used for Sholl analysis described above. None of these measures showed significant differences at 13 weeks (Figure 3A and 3C, and Table 3). Dendrite length as a function of distance from the soma closely resembled the results from Sholl analysis at 16 weeks (Figure 3B and 3D, Table 4). There was a main effect of distance×genotype (*p*<0.0001) and a distance×genotype×exposure interaction (*p*<0.019). Spine number as a function of distance from the soma also showed distance×genotype (*p*<0.01) and distance×genotype×exposure (*p*<0.0001) effects at 16 weeks (Table 4). There were significant distance×genotype (*p* = 0.032) and distance×exposure interactions (*p* = 0.015) at 16 weeks for spine density as a function of distance from the soma as well (Table 4). These data corroborate the findings by Sholl analysis, and further demonstrate that disease-toxicant interaction effects between HD and Mn exposure become apparent 3 weeks post-exposure.

### Onset of striatal neuropathology occurs by 16 weeks in YAC128 mice

**Figure 4:**
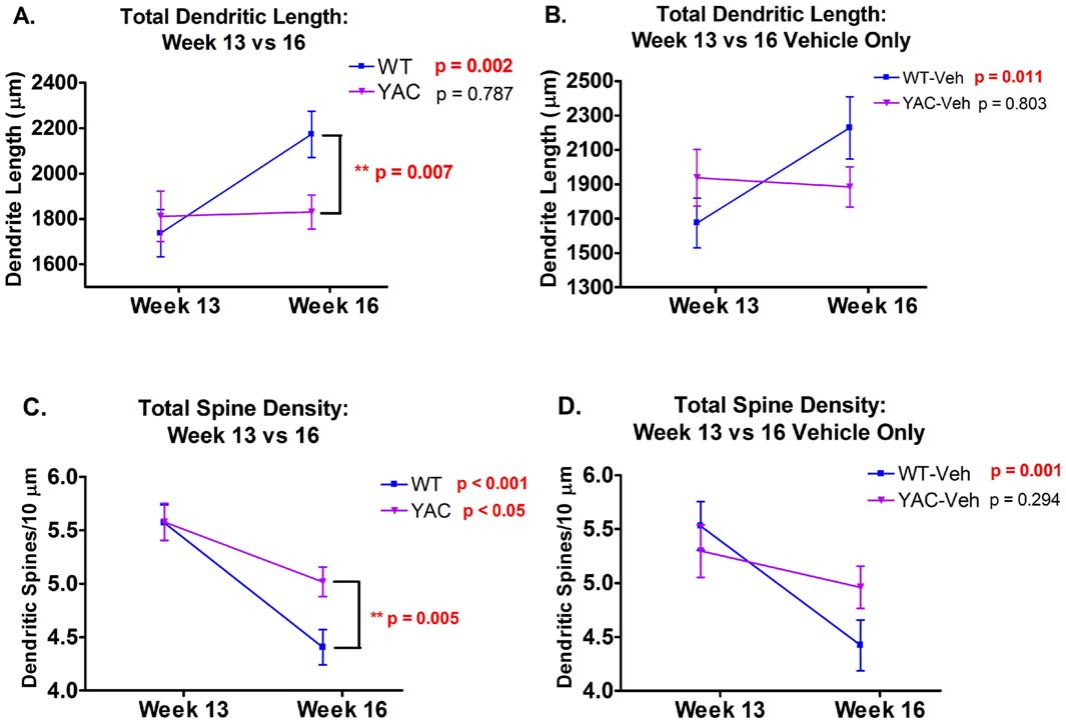
Onset of striatal neuropathology in YAC128 mice. **A**) Total dendritic length of WT mice (Veh + Mn) increased from week 13 to week 16, but was unchanged in YAC128 mice (Veh + Mn). **B**) Total dendritic length increased in WT-Veh mice from week 13 to week 16, but was unchanged in YAC128-Veh mice. **C**) Total spine density decreased from 13 to 16 weeks in both WT mice (Veh + Mn) and YAC128 mice (Veh + Mn). YAC128 mice had significantly higher total spine density than WT mice at 16 weeks. **D**) Total spine density decreased in WT-Veh mice from 13 to 16 weeks, but there was no significant change in total spine density of YAC128-Veh mice. Week 13: n = 4–5 mice per group, 4–6 neurons/animal. Week 16: n = 5 mice/group, 4–6 neurons/animal. Average values +/− SEM; p-values from *post-hoc* t-tests are on each graph, significant differences within each genotype between weeks 13 and 16 are indicated in bold, significant differences within a given time point between genotypes are indicated by (**).

Comparing total dendritic length and total spine density across age revealed that there was a significant effect of age for both neuron measures and an age×genotype interaction for total dendritic length (Table 5). While there was a significant increase in total dendritic length in WT (Veh + Mn) MSNs from 13 to 16 weeks (*p* = 0.002), there was no significant difference in YAC128 (Veh + Mn) MSNs at these time points (Figure 4A). *Post-hoc* t-tests revealed a significant difference in total dendritic length between WT and YAC128 groups at week 16 (*p* = 0.007). Based on this novel result, we decided to directly compare WT-Veh and YAC128-Veh mice (Figure 4B). There was a significant age×genotype interaction for total dendritic length between WT-Veh and YAC128-Veh (*p*<0.05). Total dendritic length increased in WT-Veh mice from 13 to 16 weeks (*p* = 0.011), while YAC128-Veh mice showed no significant difference in total dendritic length across age. Similarly, total spine density (Figure 4C) decreased from week 13 to 16 in both genotypes (p<0.05). However, at 16 weeks total spine density was higher in YAC128 MSNs compared to WT MSNs (p = 0.005). Again comparing just WT-Veh and YAC128-Veh mice total spine density decreased from 13 to 16 weeks in WT-Veh MSNs (p = 0.001), but not in YAC128-Veh animals (Figure 4D). The absence of an age-dependent increase in total dendritic length or an age-dependent decrease in total spine density of MSNs in YAC128 mice is the earliest sign of neuropathology identified to date in this model of HD.

**Table 5:** Multivariate ANOVA statistics for total dendritic measures across age.

	Genotype	Exposure	Age	Genotype×Exposure	Age×Genotype	Age×Exposure	Age×Genotype×Exposure
Total Dendritic Length	F(1,202) = 2.194	F(1,202) = 0.909	F(1,202) = 5.843	F(1,202) = 1.149	F(1,202) = 4.131	F(1,202) = 0.014	F(1,202) = 1.103
	p = 0.140	p = 0.342	**p = 0.017**	p = 0.285	**p = 0.043**	p = 0.906	p = 0.295
Total Spine Density	F(1,202) = 3.677	F(1,202) = 1.272	F(1,202) = 28.812	F(1,202) = 0.941	F(1,202) = 3.522	F(1,202) = 0.785	F(1,202) = 0.269
	p = 0.057	p = 0.261	**p<0.0001**	p = 0.333	p = 0.062	p = 0.377	p = 0.604
Total Spine Number	F(1,202) = 0.002	F(1,202) = 0.041	F(1,202) = 0.528	F(1,202) = 0.406	F(1,202) = 0.299	F(1,202) = 0.072	F(1,202) = 0.719
	p = 0.965	p = 0.840	p = 0.468	p = 0.525	p = 0.585	p = 0.788	p = 0.397
Soma Area	F(1,202) = 0.414	F(1,202) = 0.001	F(1,202) = 0.104	F(1,202) = 0.067	F(1,202) = 3.197	F(1,202) = 0.630	F(1,202) = 0.069
	p = 0.521	p = 0.981	p = 0.748	p = 0.795	p = 0.075	p = 0.428	p = 0.793
Total Nodes	F(1,202) = 0.005	F(1,202) = 1.040	F(1,202) = 0.001	F(1,202) = 2.51	F(1,202) = 3.197	F(1,202) = 0.002	F(1,202) = 0.181
	p = 0.944	p = 0.309	p = 0.974	p = 0.115	p = 0.075	p = 0.961	p = 0.671
Total Ends	F(1,202) = 0.016	F(1,202) = 0.649	F(1,202) = 0.021	F(1,202) = 1.757	F(1,202) = 0.075	F(1,202) = 0.032	F(1,202) = 0.369
	p = 0.901	p = 0.421	p = 0.886	p = 0.186	p = 0.784	p = 0.859	p = 0.544

Given the onset of the HD phenotypes between 13 and 16 weeks by total gross morphological characteristics, we next sought to determine if Sholl analyses of neuron morphology as a function of age would corroborate the onset of pathology or reveal additional Mn-exposure or genotype by Mn-exposure interaction effects. Indeed, significant distance×age×genotype interactions for dendritic branching, dendrite length and spine number were found, as well as significant effects of distance×age for dendritic branching, dendrite length and spine density; and significant distance×genotype×exposure effects for dendritic length and spine number (Table 6). *Post-hoc* analysis revealed that the genotype effect was driven by a significant increase in branching complexity between 13 and 16 weeks in WT mice (p<0.05). Importantly, there was no significant difference between 13 and 16 week branching complexity in YAC128-Veh MSNs. Therefore, changes in dendritic branching that normally occurred in WT-Veh mice *did not* occur in YAC128-Veh mice, confirming an onset of neuropathological changes between 13 and 16 weeks of age in this model. Similar results were found for dendrite length as a function of distance from the soma with WT mice having longer dendrite lengths per shell at 16 weeks compared to 13 weeks (p<0.05), whereas YAC128-Veh mice did not show this effect. Finally, the genotype×exposure interaction was validated in this expanded statistical model that included all 13 and 16 week data points (Table 6). Together these data corroborate that subtle neuropathological changes in MSN morphology commence as early as 16 weeks of age and that the disease-toxicant interaction between Mn and mutant *HTT* has a subtle effect on dendrite length in YAC128 mice.

**Table 6:** Repeated measures multivariate ANOVA statistics for dendritic measures as a function of distance from the soma across age.

	Distance×Genotype	Distance×Exposure	Distance×Age	Distance×Genotype×Exposure	Distance×Age×Genotype	Distance×Age×Exposure	Distance×Age×Genotype×Exposure
Sholl	F(39,7878) = 1.196	F(39,7878) = 0.329	F(39,7878) = 3.831	F(39,7878) = 1.257	F(39,7878) = 1.714	F(39,7878) = 0.415	F(39,7878) = 0.773
	p = 0.188	p = 1.000	**p<0.0001**	p = 0.132	**p = 0.004**	p = 1.000	p = 0.843
Dendritic Length	F(39,7878) = 1.222	F(39,7878) = 0.268	F(39,7878) = 3.932	F(39,7878) = 1.542	F(39,7878) = 2.355	F(39,7878) = 0.642	F(39,7878) = 0.679
	p = 0.162	p = 1.000	**p<0.0001**	**p = 0.017**	**p<0.0001**	p = 0.959	p = 0.937
Spine Number	F(39,7878) = 0.568	F(39,7878) = 0.248	F(39,7878) = 1.010	F(39,7878) = 1.917	F(39,7878) = 1.922	F(39,7878) = 0.836	F(39,7878) = 1.374
	p = 0.986	p = 1.000	p = 0.309	**p = 0.0005**	**p<0.0005**	p = 0.755	p = 0.061
Spine Density	F(17,3434) = 0.748	F(17,3434) = 1.104	F(17,3434) = 6.458	F(17,3434) = 0.783	F(17,3434) = 1.041	F(17,3434) = 1.219	F(17,3434) = 1.464
	p = 0.755	p = 0.343	**p<0.0001**	p = 0.715	p = 0.409	p = 0.240	p = 0.098

### Striatal DA levels are altered by Mn exposure and genotype at 16 weeks

**Figure 5:**
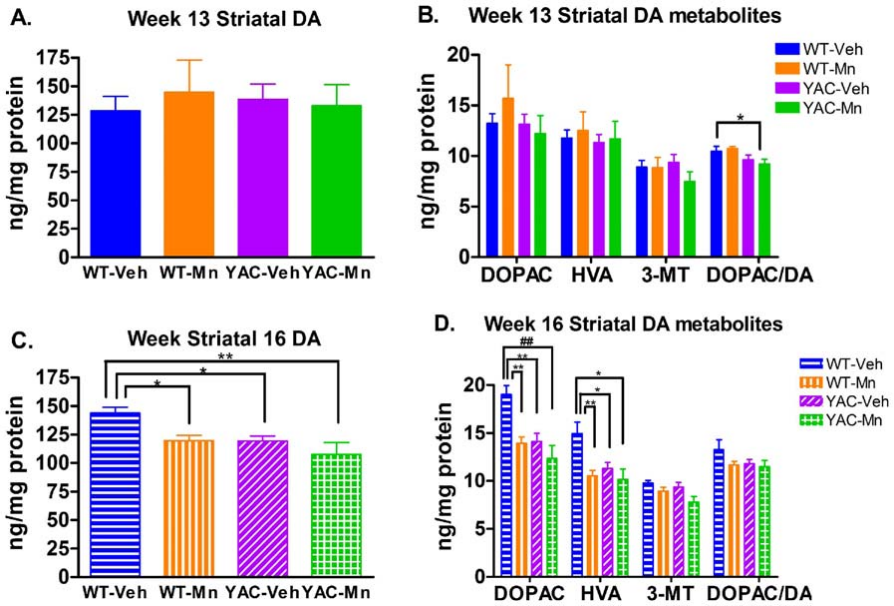
Striatal DA and metabolites are affected at week 16 by Mn exposure and genotype. **A**) There are no significant differences in striatal DA levels at week 13. **B**) There is no difference between striatal levels of DOPAC or HVA at week 13, however there is a significant main effect of genotype on ratio DOPAC/DA. The turnover rate of DA is significantly lower in YAC128-Mn than WT-Mn. **C**) At week 16 there is a significant effect of genotype and exposure on striatal DA levels. WT-Mn, YAC128-Veh and YAC128-Mn have lower levels of striatal DA than WT-Veh mice. **D**) There are also significant genotype and exposure effects on DA metabolites DOPAC and HVA at week 16. Levels of striatal DOPAC and HVA in WT-Mn, YAC128-Veh and YAC128-Mn are significantly lower than in WT-Veh mice. Monoamine neurotransmitter and metabolite means (week 13 n = 4–6 and week 16 n = 3–8) are graphed +/−SEM. Significance by *post-hoc* t-test *p<0.05, **p<0.01 and ^##^p<0.005.

Striatal DA and DA metabolites, which have not been previously reported in the YAC128 mice but have been reported to change upon Mn exposure, were measured in the striatum at 13 and 16 weeks by HPLC. At 13 weeks, DA (Figure 5A) and its metabolites (Figure 5B), 3,4-dihydroxyphenylacetic acid (DOPAC), homovanillic acid (HVA) and, 3-methoxytyramine (3-MT), were not significantly different between genotype and exposure groups although the DA turnover rate, measured by the DOPAC/DA ratio (Figure 5B), was significantly affected by genotype (*p*<0.05). DA turnover was reduced in YAC128-Mn compared to WT-Mn mice (p<0.05). Overall, at 13 weeks there were no changes in striatal DA levels, but the DA turnover rate was significantly affected by genotype. By 16 weeks there were significant exposure and genotype effects on DA levels in the striatum. Significant main effects of exposure occur for DA (Figure 5C, *p*<0.05), DOPAC (Figure 5D, *p*<0.05), and HVA (Figure 5D, *p*<0.005) levels in the striatum. There were also significant main effects of genotype on DA (*p*<0.03) and its metabolites DOPAC (*p*<0.02) and HVA (*p*<0.02).

Striatal DA and its metabolites exhibited genotype and exposure effects at 16 weeks. DA levels (Figure 5C) were reduced by Mn exposure in WT mice (p<0.05). YAC128-Veh mice had decreased striatal DA levels (p<0.05) that were indistinguishable from WT-Mn levels. Unlike in WT mice, Mn exposure failed to decrease DA levels in YAC128 mice. Striatal DA levels in YAC128-Mn mice were similar to YAC128-Veh and WT-Mn levels. DA metabolites DOPAC and HVA (Figure 5D) were similarly affected by genotype and Mn exposure. Mn exposure reduced DOPAC and HVA levels in WT mice (p<0.01). Similarly, YAC128-Veh mice had DOPAC and HVA levels reduced by approximately the same degree as WT-Mn versus WT-Veh (p<0.05). DA metabolites in YAC128 mice were not further affected by Mn exposure with DOPAC and HVA levels were similar between YAC128-Mn, YAC128-Veh and WT-Mn mice. The DOPAC and HVA metabolite, 3-MT, was not significantly different in any group at 16 weeks (Figure 5D). The DOPAC/DA ratio was also unaltered at 16 weeks (Figure 5D). Analysis of striatal DA neurochemistry across time points revealed an age×exposure interaction for DOPAC (F_(1,37)_ = 4.28, *p*<0.05) and HVA (F_(1,37)_ = 5.12, *p*<0.05). These data revealed an effect of Mn exposure and genotype on the nigrostriatal dopaminergic system that only occurred at the later time point. Overall, at 16 weeks, Mn exposure reduced striatal DA and metabolites specifically only in WT animals. Mutant *HTT* reduced striatal DA and metabolites as well, relative to WT-Veh. However, Mn exposure failed to show an effect on striatal DA and its metabolites in YAC128 mice.

## Discussion

This study identifies novel *in vivo* correlates of a recently discovered gene-environment interaction between the disease-causing allele of *HTT* and Mn exposure revealed by analysis of striatal neurochemistry and MSN dendritic architecture. Additionally, we identified the age of onset for striatal dendritic neuropathology and neurochemical phenotypes in the YAC128 HD mouse model to be between 13 and 16 weeks of postnatal life, approximately corresponding to the age of onset for motor phenotypes [38], [45], [46].

### Onset of YAC128 striatal neuropathology

The full-length YAC128 HD model was utilized for these studies because the onset of motor symptoms and neuropathology recapitulate many aspects of the human disease [38], [47]. Degenerative changes in MSN morphology have been found in multiple HD models [48], [49], [50], [51], however, striatal MSN morphology has only been reported for YAC128 mice at one month of age with no alterations identified [38], [52]. Neuron morphology studies of striatal MSNs in R6/1[48], R6/2 [50], HD48 and HD89 [49] mice showed decreases in spine density across branch order that were only evident in symptomatic mice [50]. Milnerwood et al found no morphological differences in striatal MSNs in YAC128 mice at 4 weeks of age [52]. We also detected no morphological or neurochemical associated abnormalities in YAC128-Veh mice at 13 weeks (Figures 3, 4, and 5, and Figure S1). Indeed our measurements of total dendritic length and total spine density for the 13 week WT-Veh and YAC128-Veh groups are nearly identical to values reported by Milnerwood et al at 4 weeks of age (Figure S1) [52]. Morphological and neurochemical changes in the striatum of the YAC128 mice were observed at 16 weeks. YAC128 mice failed to show an increase in total dendritic length and dendritic branching that was apparent in WT mice from 13 to 16 weeks (Figure 4) indicating the onset of MSN neuropathology in YAC128 mice between this window. YAC128 mice also failed to show a significant decrease in total spine density at 16 weeks (Figure 4). These data strongly suggest that the onset of dendritic pathophysiology in MSNs of the YAC128 model is between 13 and 16 weeks of age – coinciding with the reported onset of hyperkinetic motor behavior by open field [38]. Changes in neuron morphology of similar magnitude in other brain regions have been reported to affect brain function [53], [54]. Furthemore, the changes in neuron morphology that we observed are similar to reports in the literature examining the influence of age on neuron morphology [54], [55], [56], [57], [58], [59], [60]. For example, there is a 15% difference in dendritic length by sholl analysis between 2 to 18 months in rat frontal cortex [55]. Indeed, small differences in dendritic arborization or dendritic spine density significantly alter neuron connectivity and excitability, causing differences in signal propagation [61], [62], [63], [64], [65]. Thus, although the differences in neuron morphology we report here may appear small, the functional consequences could be significant [66], [67], [68], [69].

The morphological changes that we observed in YAC128-Veh mice at 16 weeks (Figures 3 and 4, and Figure S1) may be related to alterations in striatal neurotransmitter content. At 16 weeks YAC128-Veh mice had significantly lower levels of DA and its metabolites, DOPAC and HVA, (Figure 5) compared to WT-Veh mice. Reduced striatal DA levels and disturbances in dopaminergic signaling have also been observed in the R6/1 [70] and R6/2 mouse models of HD [71], [72], [73], [74]; however striatal DA levels were only altered in L-DOPA treated YAC128 mice [75].

### Mn exposure is associated with changes in striatal DA neurochemistry of WT mice

There was a significant decrease in striatal DA, HVA and DOPAC (Figure 5) at week 16 in WT-Mn mice, which was not present at 13 weeks. Previous studies using different Mn exposure paradigms have found similar alterations in striatal DA levels [25], [27], [28], [29], [30], [76], [77], although conflicting studies have also been reported [26], [31], [32], [78], [79], [80]. The alterations in striatal DA levels at 16 weeks may be directly related to the morphologic changes in MSNs at the same age. For example, the decreased dendritic complexity could either arise from or cause the reduced striatal DA. While more detailed studies are needed to define cause-effect relationships between neurotransmitters and the dendritic ultrastructure, our data are consistent with other reports in the literature of Mn neurotoxicity leading to dysfunction of the nigrostriatal DA system [34], [81], [82].

### HD-Mn interactions are associated with alterations in striatal neurochemistry and MSN morphology

YAC128 mice showed less striatal Mn accumulation one day after final Mn exposure at 13 weeks compared to WT littermates (Figure 1). These data replicate findings from a previous study that utilized GFAAS to measure striatal Mn levels in similarly exposed YAC128 mice [37]. There are several potential mechanisms that may influence the differential Mn accumulation between genotypes; mutant *HTT* may interfere with import of Mn into the striatum or increase efflux of Mn from the striatum. Additional research is needed to determine the exact mechanism for differential striatal Mn accumulation between YAC128 and WT mice.

MSN morphology was significantly altered at 16 weeks by the combination of the HD genotype and Mn exposure (Figures 3 and 4, and Tables 2 and 4). No differences in MSN morphology were observed at 13 weeks when Mn levels were elevated (Figures 3 and 4, and Tables 1 and 3). This is not entirely surprising because complex changes in MSN morphology, such as branching complexity, would not be expected to occur such a short time following exposure to a toxicant. Studies utilizing the DA neurotoxicant 6-hydroxydopamine (6-OHDA) have shown that changes in MSN morphology occur three weeks post-exposure [83], [84].

Mn exposure had significant effects on striatal DA neurochemistry in YAC128 mice (Figure 5). Changes in striatal neurochemistry following Mn exposure did not affect every neurotransmitter; for example, serotonin was unaffected by Mn at both 13 and 16 weeks (data not shown) and exposure to Mn did not reduce striatal DA levels in YAC128-Mn mice below YAC128-Veh or WT-Mn levels at 16 weeks (Figure 5). We hypothesize that striatal DA levels were not further reduced in YAC128-Mn mice because these animals accumulate less striatal Mn than WT-Mn mice, thus the decreased net Mn uptake in the striatum may ameliorate this toxicity in the YAC128 mice – consistent with our main hypothesis that the HD mutation would suppress Mn toxicity in the striatum.

The gene-environment interaction between mutant *HTT* and Mn, however, is complex. While for the DA related phenotype it appears the YAC128 animals may suppress Mn-dependent changes, the same does not appear to be true for dendritic morphology phenotypes. Indeed, YAC128 animals showed increased sensitivity to Mn exposure for several outcome measures of our Golgi analysis – consistent with our alternative hypothesis. Several morphological changes in striatal MSNs at 16 weeks including dendritic branching, dendritic length and spine number were all more severely affected in the YAC128 animals exposed to Mn versus the WT animals (e.g. Figure 3). Further studies need to be carried out to better understand this recently discovered disease-toxicant interaction. Nonetheless, our data strongly suggest that Mn exposure has the potential to influence HD onset or progression.

### Conclusions

We set out to determine if Mn, mutant *HTT* or the combination of the two would alter striatal neurochemistry or MSN morphology. Overall, the answer is yes – but the direction and degree of the effects and interactions were varied and depended upon the specific phenotype being examined. Mn exposure has chronic effects on neurochemistry. Striatal DA is significantly reduced at 16 weeks in WT-Mn mice. However, we failed to detect morphological alterations in MSN dendrites of WT mice exposed to Mn at either time point. We identified the onset of alterations in striatal neurochemistry and MSN dendritic neuropathology at 16 weeks postnatal in YAC128 mice. The neurochemical changes that we identified may promote NMDAR excitotoxicity in the YAC128 mice. Finally, we identified *in vivo* correlates of a disease-toxicant interaction as anticipated by our previously reported *in vitro* interactions [37], [40]. However, the finding of both restorative (DA neurochemistry) and degenerative effects (dendritic morphology changes) by Mn exposure on the early YAC128 mutant phenotypes suggests caution should be taken in determining the appropriate nutritional guidelines and environmental exposures to Mn for HD patients. This study presents the first insight into morphological changes in MSN in YAC128 mice, however we do not address the functional significance of these changes. Further investigation needs to be completed to determine the effect of the HD-Mn interaction on disease progression both at the neuroanatomical and behavioral level, as well as to gain insight into targets and mechanisms of disease modification.

## Supporting Information

Figure S1 **Total dendritic length and total number of endings are reduced while total spine density is increased in YAC compared to WT mice at 16 weeks.** **A**) Total dendritic length is unchanged in WT and YAC mice at week 13. **B**) Total dendritic length is reduced in YAC vs WT mice revealing a significant main genotype effect at 16 weeks. **C**) Total spine density is unchanged across exposure groups at week 13. **D**) The increased total spine density in YAC vs WT mice reveals a significant main effect of genotype at week 16. **E**) No significant difference in total number of endings was observed at 13 weeks. **F**) Total number of endings were reduced in YAC128-Veh vs WT-Veh mice revealing a significant genotype effect. Week 13: n = 4–5 mice per group, 4–6 neurons/animal. Week 16: n = 5 mice/group, 4–6 neurons/animal. Error bars indicate SEM. Significant differences by post-hoc t-test indicated by *p<0.05.
(TIF)
