# Supplementary figures and images for: Disease-Toxicant Interactions in Manganese Exposed Huntington Disease Mice: Early Changes in Striatal Neuron Morphology and Dopamine Metabolism
Source: PLoS One. 2012 Feb 17;7(2):e31024. doi: 10.1371/journal.pone.0031024 (PMC3281892; doi:10.1371/journal.pone.0031024)

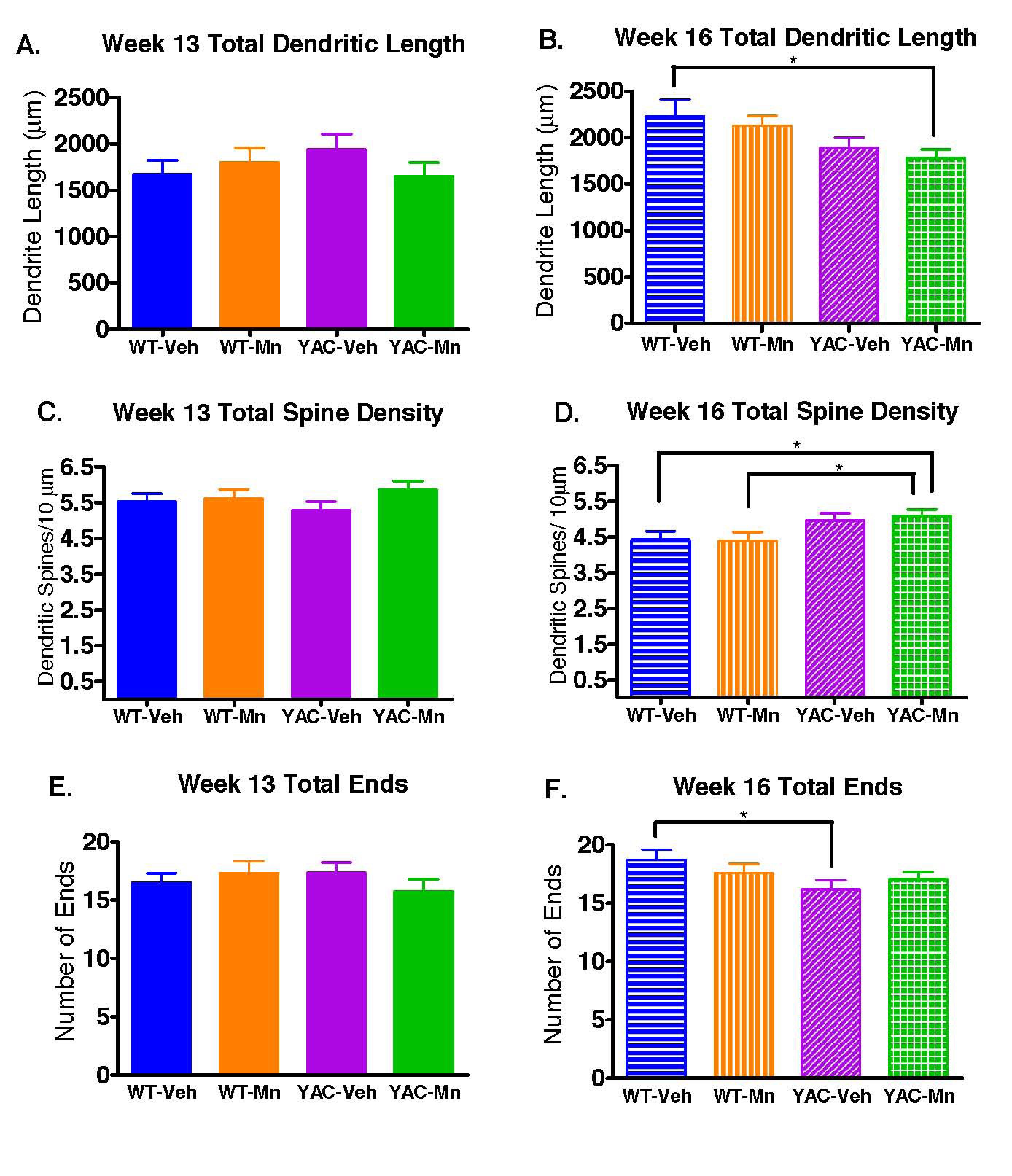

Supplement: Figure S1 — Total dendritic length and total number of endings are reduced while total spine density is increased in YAC compared to WT mice at 16 weeks. A) Total dendritic length is unchanged in WT and YAC mice at week 13. B) Total dendritic length is reduced in YAC vs WT mice revealing a significant main genotype effect at 16 weeks. C) Total spine density is unchanged across exposure groups at week 13. D) The increased total spine density in YAC vs WT mice reveals a significant main effect of genotype at week 16. E) No significant difference in total number of endings was observed at 13 weeks. F) Total number of endings were reduced in YAC128-Veh vs WT-Veh mice revealing a significant genotype effect. Week 13: n = 4–5 mice per group, 4–6 neurons/animal. Week 16: n = 5 mice/group, 4–6 neurons/animal. Error bars indicate SEM. Significant differences by post-hoc t-test indicated by *p<0.05. (TIF) [file pone.0031024.s001.tif]
